# Early Childhood Education and Care Access for Children from Disadvantaged Backgrounds: Using a Framework to Guide Intervention

**DOI:** 10.1007/s10643-019-01002-x

**Published:** 2019-11-13

**Authors:** Judith Archambault, Dominique Côté, Marie-France Raynault

**Affiliations:** Léa Roback Research Centre on Social Inequalities, Université de Montréal, 1301, rue Sherbrooke Est, Montreal, QC H2L 1M3 Canada

**Keywords:** Childcare, Access, Equity, Framework

## Abstract

Early childhood education and care (ECEC) can have substantial beneficial effects on overall child development and educational success for children from disadvantaged backgrounds. Unfortunately, it is well documented that they are underrepresented in quality ECEC programs. In order to improve access to quality ECEC, it is important to understand the factors leading to these inequities. This paper is based on a synthesis of published literature on interventions aimed at improving access to ECEC. We propose a framework identifying the spectrum of factors influencing access to quality ECEC for disadvantaged populations. We also present, in the context of our proposed framework, different interventions that have been taken to improve access to ECEC opportunities for children from low socioeconomic and/or new immigrant backgrounds. We believe that the framework proposed in this paper serves not only as a framework by which to understand the overlapping processes, factors, and stages affecting access to ECEC, but also as a model to help decision makers coordinate their efforts and maximize their impact towards more equity in access to quality early childhood education.

## Background

Early childhood education and care (ECEC) can have substantial beneficial effects on overall child development and educational success for children from disadvantaged backgrounds, making ECEC a powerful strategy to reduce child development inequities (Burchinal et al. 2010; Burger 2010; Vandenbroeck et al. 2014). Another beneficial effect of ECEC is allowing mothers to go back to work and therefore raising family income and fostering economic development (Fortin et al. 2012; Raynault and Côté 2016). For these reasons, public policies investing in early childhood are one of the best investments one can make in human capital (Heckman 2006; Organisation for Economic Co-operation and Development 2015).

Different configurations of ECEC exist in Quebec, Canada, depending on the funding source (private or public) and the setting (private home or facility). “Centres de la petite enfance” (CPEs) are non-profit organisations or cooperatives subsidised by the Quebec government. Among all configurations of ECECs in Quebec, CPEs usually offer better quality environments and services to fight school preparedness inequalities, particularly for children from more disadvantaged backgrounds (Drouin et al. 2004; Guay and Laurin 2016; Japel et al. 2005).

Unfortunately, it is also well documented, in Quebec as well as in other western countries, that children from disadvantaged backgrounds are underrepresented in quality ECEC programs such as CPEs (Guay et al. 2015; Laurin et al. 2015; Raynault et al. 2010; Vandenbroeck et al. 2008). In Montreal, proportionally fewer children from disadvantaged backgrounds have been found to attend a CPE (exclusively, or at any point in their lives) compared to their more advantaged peers (21% vs. 37% and 35% vs. 55%) (Guay et al. 2015). In order to improve access for disadvantaged children to quality ECEC programs, it is important to understand the factors leading to these inequities.

While there is a growing body of literature considering the factors affecting equitable access to ECEC, the factors have typically being considered in isolation. Recently, Vandenbroeck and Lazzari (2014) began considering the interplay between multiple factors and proposed a framework for inclusive policy and practices at three different levels: governance, management of services, and parents (Vandenbroeck and Lazzari 2014). Meanwhile, in the literature about equitable access to healthcare, an integrated conceptual framework describing the complex comprehensive and dynamic concept of access to healthcare has been proposed by Lévesque et al. (2013). We believe this framework can be applied to access to ECEC in order to adopt a more integrated approach to understand and develop interventions to reduce inequity of access to ECEC programs. The goal of this paper is twofold. First the Lévesque and colleagues’ framework was adapted and the spectrum of factors influencing access to quality ECEC for disadvantaged populations identified. Second, different interventions that have been taken to improve access to ECEC for children from low socioeconomic and/or new immigrant backgrounds found in the existing literature were gathered and presented in the context of our proposed framework. The framework brings individual interventions into a coherent and comprehensive structure that recognizes the importance of the complementarity of interventions between different partners working towards a common goal. As such it serves not only as a framework by which to understand the overlapping processes factors and stages affecting access to ECEC, but also as a model to help decision makers coordinate their efforts and maximize their impact.

## Method

The first step towards the writing of this paper was a synthesis of published literature on interventions aimed at improving access to ECEC. Several sources were used to identify research related to access to ECECs that had been published from 1997 to 2017. These sources were 14 databases from the Proquest plateform as well as the Famili@ quebec research database and the Canadian Public Health Agency website. The following terms were used when searching these sources: child and early children education center (daycare, childcare, nursery, kindergarten), characteristics of the population (low socioeconomic status, vulnerable, immigrant, disadvantaged, in need, at risk, poor, poverty), access (access, availability, cost, price, accessibility, use) and interventions (project, action, program, measure, intervention, policy, service). References relevant to the study that did not appear in results obtained as described above but that were found in articles that were, were also included in the analysis.

Papers were screened and selected if they included a description of an intervention aimed at improving ECEC access for disadvantaged children and families. Papers solely referring to the benefits of ECEC or the barriers of access without proposing solutions and avenues towards overcoming such barriers were not included in the review. At the end of the process 19 articles were selected and reviewed.

## Results

### Adapting Levesque et al. ’s Framework to ECEC Access

**Fig. 1:**
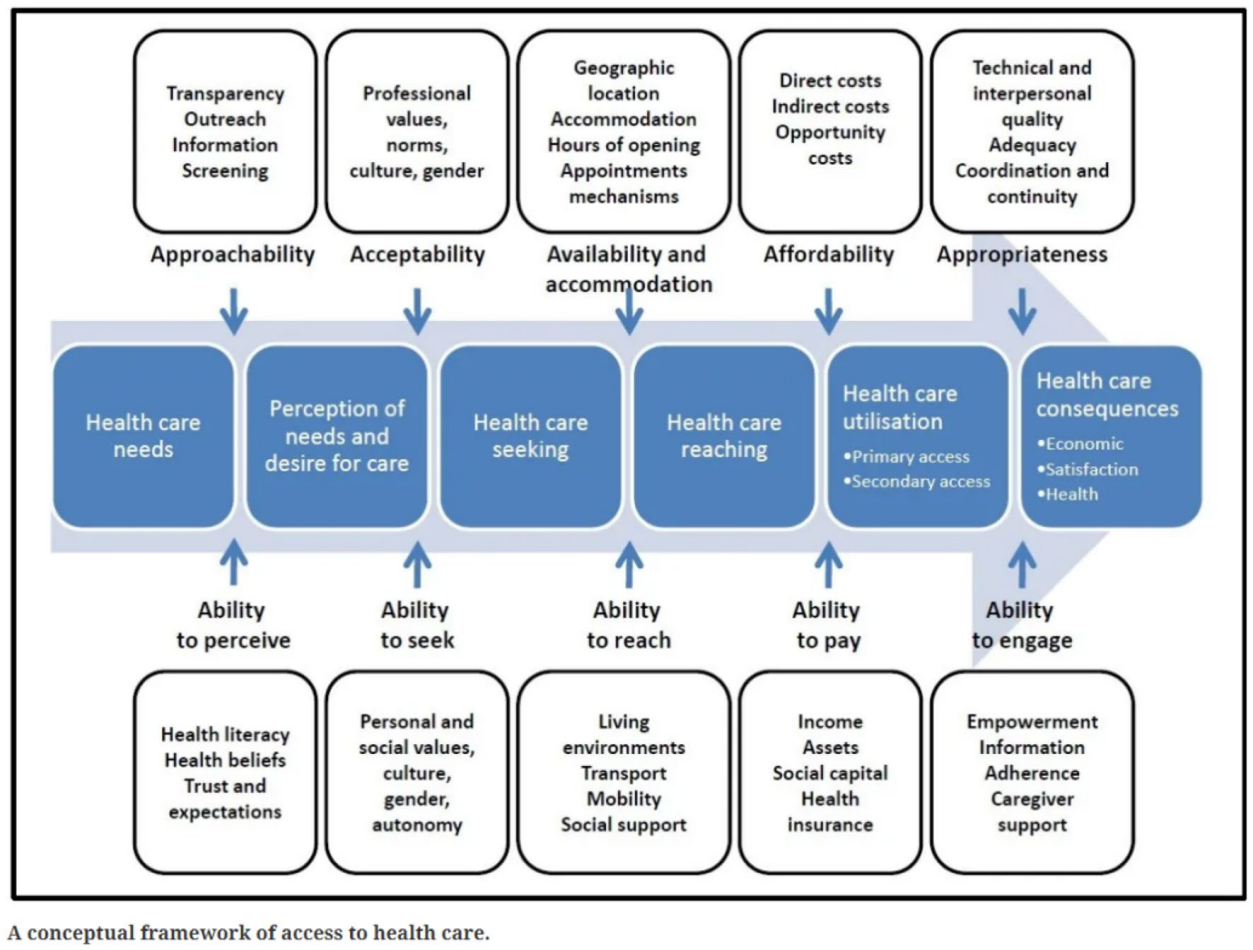
A conceptual framework of access to health care by Levesque et al. (2013)

The goal of Levesque et al.’s framework is to bring an integrated approach to understanding access to health care. They identify five “supply-side” dimensions of accessibility that interact with five corresponding “demand-side” abilities of populations that influence access to care at each stage along a continuum from need for care to benefiting from it (see Fig. 1) (Levesque et al. 2013).

Demand-side factors are composed of characteristics of the health system, health organizations, and providers. Supply-side factors, on the other hand, are composed of characteristics of individuals, households, and social and physical environments.

**Fig. 2:**
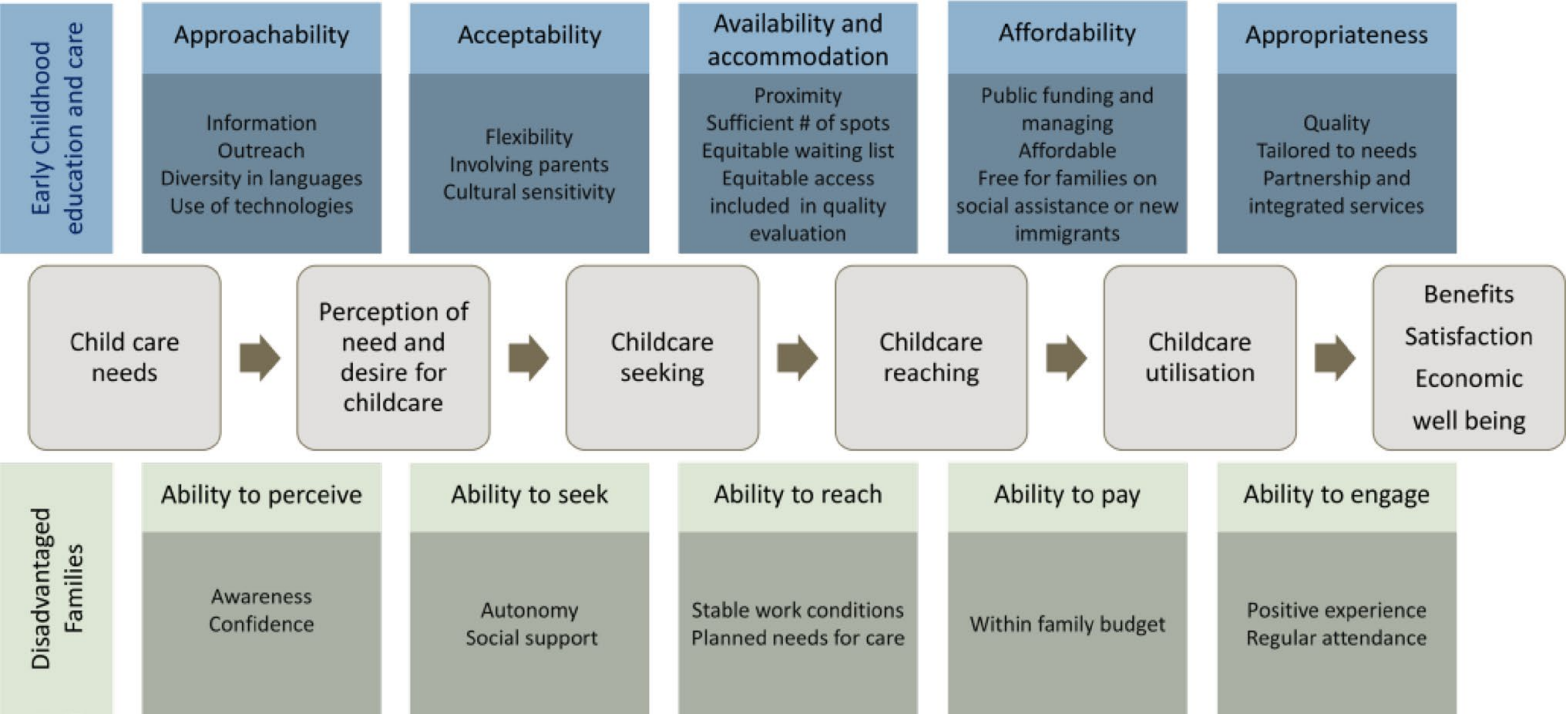
A conceptual framework of access to quality ECECs for children from disadvantaged backgrounds

Our framework is also organized along the same six stages of a continuum from need to benefit (for childcare). We consider supply-side factors to include the characteristics of ECEC programs themselves, as well as the institutional environments (funding mechanisms, geographic location, etc.). Demand-side factors, on the other hand, include the characteristics of vulnerable families as well as their cultural, physical and social environments. It is the interaction of these two sets of factors that influences access to quality ECEC at each stage. Our proposed framework is presented schematically in Fig. 2. Each of the stages and associated supply- and demand-side factors from the framework are explained below.

### Approachability and Ability to Perceive Child Care Need

In order for families to recognize their need for child care, they have first to know that ECEC programs exist as well as to hear about their benefits. Child care options should also correspond with their beliefs and values. For some families living in poverty or having recently immigrated from countries or regions where ECEC programs are rare, child care doesn’t always fit with their values, family model, or conception of a mother’s role in the household (Bigras et al. 2011; Binet 2002; Laurin et al. 2008; Lowe and Weisner 2004). Negative perceptions of child care and rumours about the poor quality of child care services can reinforce these beliefs and are significant barriers to ECEC use (Lowe and Weisner 2004). Indeed, according to a review of family public policies of 31 European countries, the higher the perceived quality of ECEC is in a country, the less access inequities there are (Van Lancker and Ghysels 2016).

#### Awareness and Confidence in Services

Awareness and information campaigns can be useful to encourage confidence in ECEC programs by publicizing their benefits, qualities, costs and registration procedures (Johnson et al. 2017).

#### Outreach

At the same time, awareness campaigns can be less effective because families living in difficult financial conditions are hard to reach through conventional channels (Halperin 2007; Pichette 2013). Therefore, some authors recommend using social and organizational connection channels (Vesely 2013). As an example, in Montreal, community organizations are able to reach young children and their families in their milieu through home visits by mothers from the same culture, mobile libraries, or through language development outreach interventions (Pichette 2013). The quality of the relationship that is developed with these families can improve the impact of interventions and favour registration into preschool programs. In a recent study about parental ECEC preferences from the Quebec provincial Family Department, health care and social care system professionals were identified as potentially efficient information sources about ECEC programs (Binet 2002). Parents who had recently immigrated to Quebec also identified immigration services agents as good potential information sources (Samson 2016).

### Diversity in Languages and Use of Technologies

In order to reduce language barriers, communicating in multiple languages is also a good practice to reach immigrant families (Johnson et al. 2017), as well as using information technologies, not only to reach these families, but also to keep in touch after they enroll in ECEC programs. This can be done by sharing day-to-day information about their children (Isik-Ercan 2012; Raynault and Côté 2013).

Through the interventions presented in this section, we can see how reaching out to underprivileged families and giving them the right information about ECEC programs calls for good communication and collaboration between intersectoral partners (Isik-Ercan 2012).

### Acceptability and Ability to Seek

In order for underprivileged families to seek out ECEC services, the services themselves must be acceptable and compatible with family needs and favor good-quality interactions between ECEC staff and families (Vandenbroeck and Lazzari 2014). It is therefore important to develop welcoming practices by adapting services to the needs of underprivileged and immigrant families.

This can be done by providing staff with training and guidance, and by having parents be part of decisional processes at the ECEC program (Association québécoise des centres de la petite enfance n.d.).

#### Flexibility and Involving Parents

Acceptability of ECEC is also favored by flexibility and the friendliness of services by, for example, allowing parents to stay in the room with their children as long as they want or offering flexible hours of attendance (Isik-Ercan 2012; Pichette 2013). These practices allow parents the opportunity to become familiar and comfortable with routines, activities and the functioning of an ECEC, before they leave their children on a regular basis.

#### Cultural Sensitivity

Cultural sensitivity and language awareness are important for strengthening the relationship between ECEC staff and immigrant mothers to develop confidence (Vesely 2013). This can be done by providing staff with training and guidance (Association québécoise des centres de la petite enfance n.d.). Hiring childcare workers from minority groups, and who are sensitive to the cultural norms of the population in question, are other examples of good practice, which favor acceptability of services in multicultural contexts (Johnson et al. 2017).

#### Autonomy and Social Support

Once the need for child care is recognized, the ability to seek services is influenced by a few factors. Social support and family autonomy favor a family’s first step towards finding a suitable service. Demanding administrative burdens (e.g., the necessity to show a birth certificate) is a barrier to enrolment for children from disadvantaged backgrounds. Because of the unavailability of identity documents or of the cost and complexity of steps for obtaining them, this can be discouraging for many families (Vesely 2013). To overcome this barrier, Vesely suggests accepting less formal documentation, such as evidence of residency for demonstrating eligibility for ECEC services (e.g. a letter from a landlord, official email sent to a member of the family, library card, etc.) (Vesely 2013).

The role of social networks is also important (Vandenbroeck and Lazzari 2014; Vesely 2013). When families hear about new spots opening in ECEC programs through neighbors, colleagues, and friends, they can have a better idea of ECEC programs that are of better quality or in line with their values. They can also learn about services in the community that can help with registration processes. Therefore, interventions to increase social integration can have a positive effect on different characteristics and stages of the access continuum, such as knowing about ECEC programs and services, favoring confidence toward ECEC, and strengthening social networks (Isik-Ercan 2012). Moreover, it is known that parents who are unemployed have fewer contacts with their peers, are less informed, and are often in a position of waiting for services to come to them (Halperin 2007), so that they are especially in need of improved social integration.

### Availability, Accommodation and Ability to Reach

It’s has been documented that there is inequity in the number of ECEC spaces available across neighborhoods (Le Blanc et al. 2011; Vandenbroeck et al. 2008). In a context where demand outstrips supply, the way waiting lists are managed can also have an impact on access. First-come-first-served management can indirectly discriminate underprivileged families who tend to subscribe to services later than more privileged families, and who have less regular work conditions making it harder to plan the moment when they are going to need childcare (Halperin 2007).

#### Sufficient Number of Spots and Proximity

A Belgian study showed an increase in geographical inequities in the number of spaces after an intervention encouraging existing ECEC programs to increase available spaces (Vandenbroeck et al. 2014). The problem of availability is therefore also a problem of geographic distribution of spaces. As such, increasing the supply of quality ECEC spaces in neighborhoods where there are fewer services should be a priority (Vandenbroeck et al. 2014).

#### Equitable Waiting List

In Belgium, ECEC directors have substantially changed their management of waiting lists after an intensive coaching to establish equitable access policies (Vandenbroeck et al. 2014). Directors who participate in the program give less weight to criteria like parental employment status and initial registration date, and more weight to social criteria like low income, ethnicity and family situation. Managers participating in the program described how they realized their practices could involuntarily discriminate against some families.

#### Equitable Access Included in Quality Evaluation

In addition to how spaces are distributed, it has also been suggested that the way in which service quality is evaluated can affect the availability of ECEC spaces for underprivileged families. In particular, Vandenbroeck et al. suggest to include in service quality evaluation, a criterion that reflects whether services are accessible to a socially diverse clientele (Vandenbroeck et al. 2008).

### Affordability and Ability to Pay

Costs of services are directly affected by public and fiscal policies targeting families and naturally also influence the degree to which disadvantaged families will use ECECs.

#### Public Funding and Managing

Available data suggests that public financing and managing of ECEC programs favor better uniformity in quality and coverage of services (Organisation for Economic Co-operation and Development (OECD) 2003), as well as reduce access inequity (Van Lancker and Ghysels 2016). Not only is the amount of money invested by governments in ECEC for service quality and access equity important, but even more important is how this money is invested. Governments who favor financing the supply of services (public networks of ECEC) have seen better results than those who favor leaving the choice to families (demand side) by giving them an amount of money to cover part of services costs (Bigras et al. 2011; Friendly 2013).

#### Affordable and Free for Families on Social Assistance or New Immigrants

The amount of money parents have to pay to benefit from services is a major determinant of access. Even a reduced contribution can still be too high for some family budgets (Raynault and Côté 2014). Similarly, it has been found that any cost reductions have to be relatively significant for families to take advantage of them (Sibley et al. 2015). Moreover, free ECEC public services for social assistance beneficiaries can be a decisional factor for families (Pichette 2013). In Norway, free time-slots for children from new immigrant families are offered in ECECs. This practice was found not only to help with progressive familiarization to services in a non-constraining way but also to speed up the learning of Norwegian by children and their parents (Raynault and Côté 2013).

Finally, free lunches and snacks increase families’ adhesion to ECECs (Pichette 2013).

### Appropriateness and Ability to Engage

When parents are satisfied with services and the services respond to their needs, attendance at ECECs becomes more regular and continuous (Vandenbroeck et al. 2008); such regularity and continuity of attendance in quality ECECs is what should be sought for better outcomes (Van Huizen and Plantenga 2018). One review has shown that maternal attitudes towards ECEC and their perceptions of the impact of ECEC change over time. Mothers were found to be more reticent to leave their children in ECEC care at first, but as they recognized the positive benefits of attendance they became more comfortable over time (Vandenbroeck et al. 2008).

#### Partnership and Integrated Services

ECEC attendance can also serve as a focal point that favors the integration of mothers within the community. As such, employment integration services or language courses associated with ECECs are promising avenues for community integration (Vesely 2013). In order to do this, Vesely (2013) suggests putting in place partnerships between ECECs and community organizations working towards literacy to offer services to parents at ECECs. Moreover, intersectoral actions (ECECs, after-school programs, parents and communities) favor the integration of immigrant families (Isik-Ercan 2012; Johnson et al. 2017).

## Discussion

In this paper, we propose a framework that brings individual interventions already reported in the literature into a coherent and comprehensive structure that recognizes the importance of the complementarity of interventions between different partners working towards a common goal. The proposed framework is based on Levesque et al.’s existing framework, which was extensively researched and is now well recognized as a central framework in improving access to healthcare. Access to healthcare and access to childcare have a lot in common. They are both characterized by a dynamic process where demand- and supply-side characteristics interact at several stages ranging from the need to use services, to the benefits derived from care. Using a framework helps identify barriers and determinants of use that can be targeted by interventions. Interventions targeting only one barrier or determinant have less chance of succeeding than a series of interventions targeting multiple elements of the framework. This is where careful planning in a multisectorial and complementary context derives its usefulness. While we think this framework is important, we also recognize that it is limited by the body of literature previously published on the subject and is only a first step in understanding and influencing the comprehensive nature of the problem.

## Conclusion

We believe that the framework proposed in this paper has the potential to improve the effectiveness of interventions aimed at improving accessibility to quality early childhood education for disadvantaged families. The framework serves not only as a framework by which to understand the overlapping processes factors and stages affecting access to ECEC, but also as a model to help decision makers coordinate their efforts and maximize their impact. As such it strengthens the role of multisectorial partners and actions towards the common goal of more equity in access to quality early childhood education by disadvantaged families. Future work will hopefully be able to build on this framework to incorporate other important and relevant factors. These are for example the nature of larger structural determinants such as social status, income inequity, and broader social and political trends.
